# Determining the Stages of Deformation and Destruction of Composite Materials in a Static Tensile Test by Acoustic Emission

**DOI:** 10.3390/ma15010313

**Published:** 2022-01-02

**Authors:** Katarzyna Panasiuk, Krzysztof Dudzik

**Affiliations:** Faculty of Engineering, Gdynia Maritime University, 81-225 Gdynia, Poland; k.dudzik@wm.umg.edu.pl

**Keywords:** composites, NDT, acoustic emission, tensile test, destruction process

## Abstract

Composite materials are used in many industries. They are construction materials that are being used more and more often, which makes it necessary to accurately identify the process of their destruction. Recent decades have resulted in an intensive increase in diagnostic tests of structures and mechanical elements. Non-destructive testing (NDT) represents a group of test methods (surface and volumetric) that provide information about the properties of the tested element without changing its structure. The method of acoustic emission (AE) is also being used more frequently. Thanks to the ability to detect and locate signal sources, as well as to perform tests during operation, it is a method that is increasingly used in industry. In this article, the acoustic emission was used to analyze the changes occurring in composite materials. Obtained parameters helped to determine the signals originating from fibre delamination, fibre cracking, etc., as well as the starting point of these changes and the stress values at which these changes occurred. The analysis of acoustic emission signals recorded during the tests helped to determine the values of amplitudes characteristic for the destruction mechanisms of considered composite materials. Signals with an amplitude in the range of 30–41 dB may indicate elastic–plastic deformation of the matrix. Signals with an amplitude in the range of 42–50 dB indicate matrix cracks with the accompanying phenomenon of fibre delamination. Signals with amplitudes greater than 50 dB indicate fibre breakage. Based on the test results, the permissible stress was determined; when exceeded, the mechanisms of damage to the structure of composite materials accumulate. This stress limit for the tested material is 70 MPa. The use of the acoustic emission method in mechanical tests may contribute to a greater knowledge of composite materials used as a construction material, as well as determine the stresses allowable for a given structure.

## 1. Introduction

Composite materials are used in many industries [1]. They are materials which are used in shipbuilding, railways, aviation, as well as in medicine and many other industries. They are construction materials that are increasingly used because of their strength and relatively low weight. These are materials, the strength of which can be shaped in any direction, which additionally makes them more attractive, but hinders the design process [2,3]. The main advantage of these materials is the easiness of obtaining complicated shapes without using complicated technology, in most cases. Composites show resistance to aging, i.e., the influence of the environment, which allows them to be used in places where steel and aluminium alloys would not work so well, e.g., reinforcement in sea water [4,5]. The most often used composites are polymers reinforced with glass fibres, i.e., glass fibre-reinforced plastics (GFRPs), as well as with the reinforcement in the form of carbon fibres, i.e., carbon fibre-reinforced plastics (CFRPs). The advantage of GFRPs and CFRPs is their ability to design and combine a variety of structures, as well as to modify mechanical and physical properties through nanofillers, which makes them an interesting material for design and research [6].

The process of destroying these materials is much more complex than metals; it involves a number of mechanisms, such as cracking at the reinforcement–matrix interface, delamination, etc. [7,8,9,10]. For example, in [11], the influence of the types of fibre hybridization on mechanical properties (interface or short-beam shear strength, three-point bending strength, and tensile strength) was experimentally presented. The deformation was shown using digital correlation. Fibre hybridization helped to obtain an increase in the shear, bending, and tensile strength properties of the composite. Moreover, it was verified that damage to the core–shell hybrid (CSH) rods, directly from the shell–core interface. In the case of uniformly dispersed hybrid (UDH) rods, the mechanical damage resulted from a carbon fibre rupture, and the interfacial bonds remained intact. The study [12] focuses on the analysis of the effect of the rod size and fibre hybridization on the shear strength under freezing and thawing conditions and the external environment. Water adsorption for random fibre hybridization increased a little compared to the core–shell mode of the fibres. Increasing the diameter of the rod had the effect of increasing the saturated water uptake time. The decrease in shear strength was due to the hydrolysis and plasticization of the resin, which in turn resulted from the absorption of water and the formation of microcracks. The tests of rods with random fibre hybridization showed the corrosion resistance of these materials.

Recent decades have resulted in an intensive increase in diagnostic tests of structures and mechanical elements. For this reason, there has been a development of methods for obtaining information from diagnostic tests in order to assess the technical condition of the device and, on this basis, to enable actions to be taken to increase its durability, reliability, and efficiency [13].

There are several methods of detecting damaged areas in composite materials [14,15]. Unlike other methods, the acoustic emission (AE) [16,17] method is very sensitive in detecting active cracks [18], even in the beginning of plastic deformation [19,20,21]. It enables monitoring and identification of signals, ranging from microscopic deformations which influence the fracture process or cracking of fibres and matrix [21,22,23]. It can be used to characterize the delamination process and provides reliable information about the onset of delamination on a microscopic as well as on a macroscopic scale [24,25]. The article [14] presents the stretching of carbon fibre tubes, which shows the dependence of force on time by the activity of acoustic emission as a function of time, thanks to which it was possible to determine the boundary separating signals from a broken fibre from undesirable secondary sources. The use of the acoustic emission method helped to identify damage mechanisms, such as matrix cracking, fibre cracking, decohesion, and detachment. In the study [19], the damage, cracking, and destruction mechanism of composites with different contents of high-filled wood fibre or recycled high-density polyethylene composites were investigated in the three-point bending test, by combining the acoustic emission (AE) technique and the scanning electron microscope (SEM). The results showed that the acoustic emission technology is extremely useful in understanding the deterioration and fracture process of these types of composites. Composite damage and cracking processes are presented in the form of three stages: initial damage, deformation of the matrix, and interfacial delamination. In the article [23], the acoustic emission method was used to detect the point responsible for the crack initiation of wood fibre or recycled high-density polyethylene composites, and the fracture resistance of these materials was assessed. The results presented in the paper show that the novel AE-based methods are more effective than conventional standard methods for characterizing the point responsible for crack initiation. With the use of the relationship of the cumulative acoustic emission events with time and load, the critical stress intensity coefficient was determined and the fracture toughness was calculated. The changes taking place in the composite structure, such as delamination, can be identified much earlier [22]. The use of mechanical tests and acoustic emission helps to detect delamination as well as other processes of destruction of composite materials [24,25]. Fibre fracture, fracture at the matrix–reinforcement interface, and matrix fracture can be identified, assessed, and analysed using parameters such as amplitude, number of events, rise time, duration, and energy of recorded AE signals [26].

The AE technique has unique advantages in the study of damage initiation and propagation, and helps to describe damage and cracking of materials together with the assessment of their resistance to these phenomena [23]. For more detailed damage monitoring, it is preferable to include an appropriate tool or signal in the analysis.

This article focuses on isolating and distinguishing signals from matrix fracture from fibre delamination and fracture at the fibre–matrix interface. These studies are a continuation and extension of previous studies on acoustic emission in composite materials [4,5]. Earlier studies analyzed the possibility of using acoustic emission to analyze changes in composite materials with polyester-glass recyclate. However, they did not focus on distinguishing the type of failure, such as matrix cracking, delamination, etc. [4]. In the article [5], the acoustic emission with the K-S entropy was used to determine the transition of the composite material from elastic to plastic state. Research on the use of acoustic emission in composite materials is carried out by many scientists; however, the material and the analysis of the obtained results are of major importance here. The results obtained as a result of acoustic emission are influenced by the type of material tested, its shape, and the test performed. The article [14] presents the results for CFRP composite bars, focusing mainly on the amplitude values for a specific type of failure. The nature of the destruction of these materials, compared to GFRP, is similar. However, this article decided to not only focus on determining the nature of the destruction and distinguishing its types, but the acoustic emission was used to determine the maximum value of the stress up to which the changes in the composite are insignificant. Designing structures based on these materials is of great importance. In addition to the static tensile test, the basic test is carried out when designing new materials. The use of acoustic emission in these tests helps to recognize this material precisely, in terms of its deformation and destruction process, as well as maximum stress values. A plate based on polyester resin without reinforcement, and also reinforced with glass fibres (GFRPs), was used for the tests. A static tensile test for composite materials was performed using an extensometer and the physical acoustics corporation acoustic emission system. By analyzing the amplitude (t) and counts (t) diagrams plotted on the material tensile diagram based on the pure resin, the signal from matrix cracking was isolated and, thus, it was marked on the graphs of the composite material with the glass fibre reinforcement.

## 2. Materials and Methods

Using the manual lamination method, a composite material was made, the matrix of which was a polyester resin, while the reinforcement was glass fiber with a weight of 350 g/m^2^. For the production, 10 layers of glass mat were used, which constituted 40% by weight of the composite material. For the production of composite materials, a mold with dimensions of 900 mm × 300 mm was used. Wax was used as a separator. Ten layers of glass mat with a weight of 450 g/m^2^ were used, which was successively saturated with resin using acetyl rollers and brushes. Due to the previous experience with making composites with this technology, 10 layers of the mat constituted 40% of the reinforcement of the entire composite, while the rest constituted −60% of resin (by weight). There was no need to add the accelerator, because it was already part of the resin. The hardener was added in the amount of 10 g per 1 kg of resin, obtaining a gel time of about 20 min. Table 1 shows the properties of the reinforcement and the matrix used in the test.

**Figure 1:**
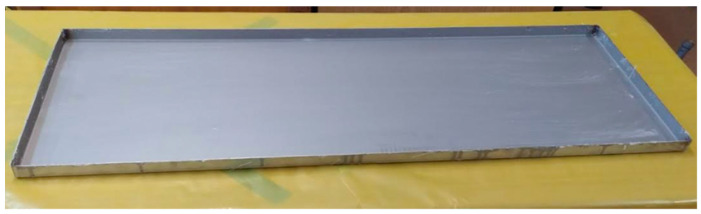
Mold for production of composite sample.

For comparison, a material based on polyester pure resin, as produced without reinforcement, containing % by weight, gave the same amount of resin as the material with reinforcement. Samples for static tensile tests were prepared in accordance with the standard (PN-EN ISO 527-4_2000P) using the water cutting method. Figure 1 shows a mold for the production of composite materials.

**Figure 2:**
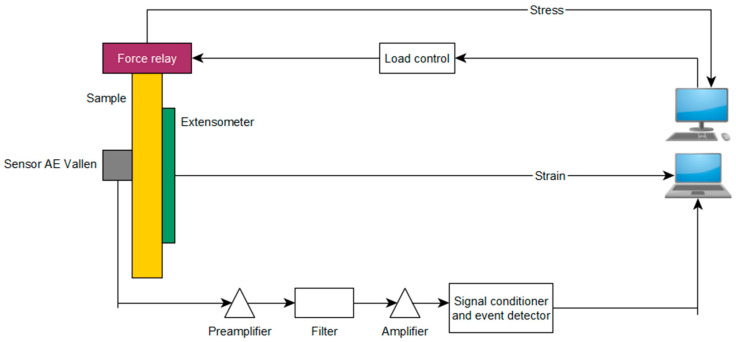
Diagram of measuring station.

The tests were carried out on the Zwick & Roell MPMD P10B universal hydraulic testing machine (ZwickRoell GmbH & Co., Ulm, Germany) with the TestXpert II software (ZwickRoell GmbH & Co., Ulm, Germany). Additionally, an Epsilon 3542 extensometer (Epsilon Technology Corporation, Jackson, USA) was used to precisely measure the elongation. For monitoring the tensile test of chosen specimens, an acoustic emission system from the Physical Acoustics Company (Physical Acoustics Corporation, Princeton, NJ, USA) was used. Diagram of the measuring stand is presented in Figure 2.

**Figure 3:**
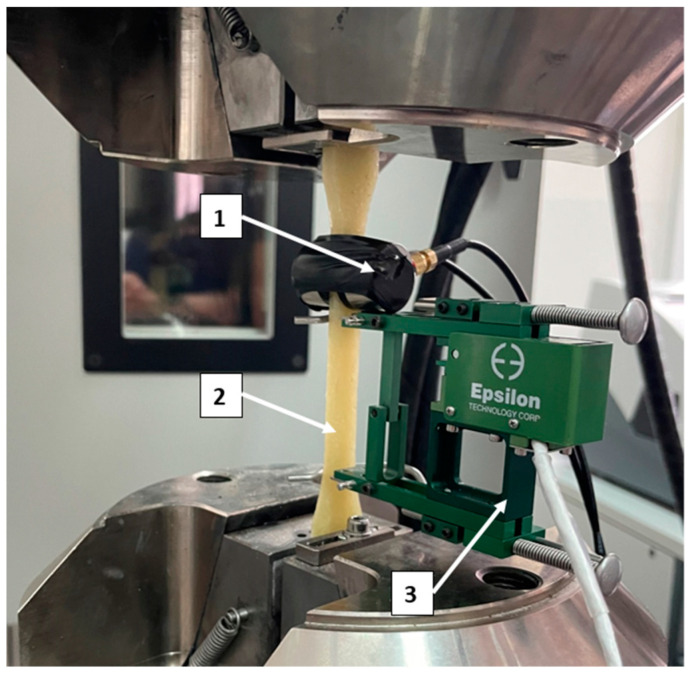
Composite sample during test: 1—AE sensor, 2—sample, 3—extensometer.

Research AE was performed using a set consisting of a single-channel recorder USB AE Node, type 1283 with bandpass 20 kHz–1 MHz, preamplifier with bandpass 75 kHz–1.1 MHz, AE-Sensor VS 150M (with a frequency range of 100–450 kHz), and a computer with AE Win for USB Version E5.30 software (Physical Acoustics Corporation, Princeton, USA) to record and analyse AE data. The tests were carried out in accordance with the applicable standards related to acoustic emission tests (PN-EN 1330-9:2017-09; PN-EN 13554: 2011E; PN-EN 15857: 2010E). Between the sensor and a surface of the specimen, a coupling fluid was used. An AE sensor was fixed to specimen by elastic tape. Figure 3 shows the sample during the test with the AE sensor and extensometer.

## 3. Results 

**Figure 4:**
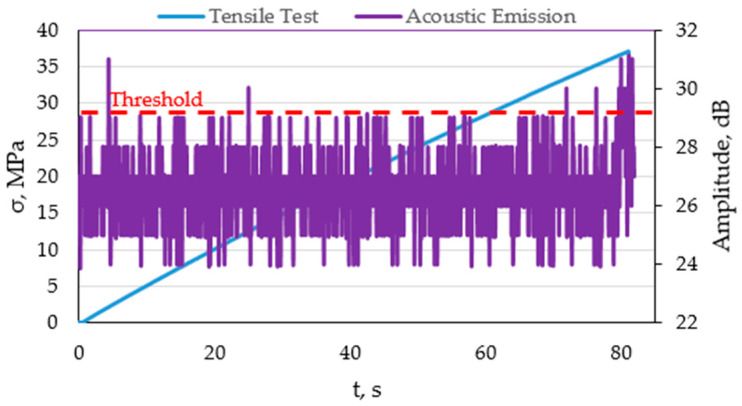
Graphs of amplitude plotted on graphs of tensile versus time for a selected sample based on pure polyester resin.

In order to assess the nature of the destruction process of the composite material, such as matrix fracture and fibre fracture, a sample based on pure polyester resin (B-Base) and glass fibre-reinforced composite (FRP) was subjected to static tensile tests. Ten samples of both materials were tested. In the base sample, the main goal was to determine the nature of the signal, the amplitude value, and the number of counts, so that these parameters could be compared with the glass fibre-reinforced composite. This made it possible to determine the load range at which matrix fracture occurs, as well as to separate these values from fibre fracture or, for example, fracture at the fibre–matrix interface. Figure 4 presents the graph of amplitude versus time plotted on the tensile graph of the base material.

Figure 4 shows a plot of amplitude versus time plotted on a tensile versus time plot. The threshold at the level of 29 dB, below which the signals constitute acoustic noise, is marked in red. The same value was found during the analysis of all samples, including those reinforced with glass fibres. Exceeding the discrimination threshold at the beginning of the test (approx. 5 s) probably comes from clamping the grip of the testing machine on the sample. Due to the fact that this material is based on pure polyester resin, and thus is brittle, from the beginning of the static tensile test, a slight elastic–plastic deformation occurs, followed by breakage. It is visible that the discrimination threshold is exceeded in about 80 s of the test, where the average value of the signal amplitude reaches about 32 dB (for chosen sample). Therefore, it can be concluded that the average range of matrix fracture for all 30 tested samples is from 30–34 dB.

**Figure 5:**
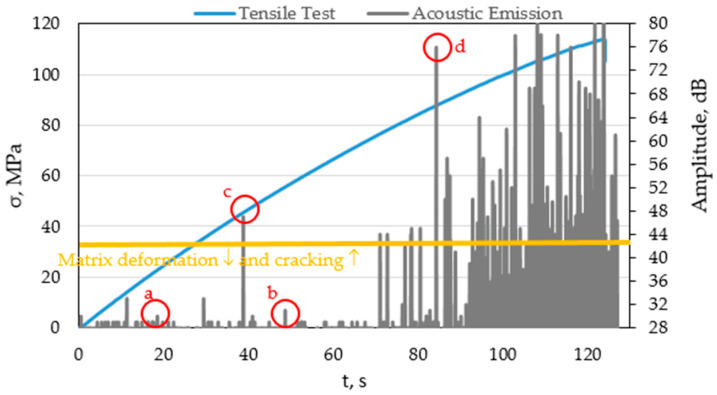
Amplitude diagrams plotted on the tensile diagrams as a function of time for a selected sample based on a glass fibre-reinforced polyester resin with the marked area of deformation and cracking of the matrix and marked points for further analysis: (a,b) matrix deformation, (c) matrix cracking, (d) cracking of fibers.

Figure 5 shows a diagram of the amplitude versus time plotted on the tensile diagram of a composite based on polyester resin, reinforced with glass fibre.

On the basis of the obtained amplitude values from the base sample based on the pure polyester resin, the area corresponding to the noise occurring during the test was determined for the sample based on the polyester resin reinforced with glass fibre. On the basis of the pure resin samples tested, it was found that, below the value of 35 MPa, we deal with matrix deformation, while above this stress-cracking.

When analysing the amplitude diagram for a glass fibre-reinforced sample, there are visible points where changes in the structure of composite materials occur, caused by warp deformation, matrix cracking, fibre cracking, and fracture at the fibre–matrix boundary.

Taking into account the stress values, the first greater amplitude jump occurs at a stress of about 45 MPa, while significant changes in the structure of the composite material occur at a stress of about 80 MPa.

Characteristic points (a, b) are marked in Figure 5 which may indicate deformation of the matrix. Their amplitude exceeds the discrimination threshold set at 31 dB. Point ‘c’, illustrating a signal with an amplitude of 48 dB, is the first one to indicate matrix fracture, which corresponds to a stress of about 45 MPa. However, this is one event that occurs precisely in this selected sample, after which there are no significant changes again to the stress value of 70 MPa. Hence, it does not significantly affect the structure of these materials and their further operation. A signal with an amplitude as shown in point ‘d’ indicates fibre breakage. After exceeding this point, corresponding to a stress of about 80 dB, the process of destroying both the fibres and the matrix of the composite takes place. After exceeding this stress value, we deal with the accumulation of various types of failure mechanisms; hence, it can be concluded that this is the maximum to which the tested material can be loaded, without a significant impact on its structure. Further tests, e.g., fatigue tests, should be carried out in order to be able to determine a 100% safe level for the structure depending on the expected loads. Due to the fact that the glass fibre-reinforced material is an elastic–plastic material, the diagram can not only distinguish matrix cracking, but also its deformation.

**Figure 6:**
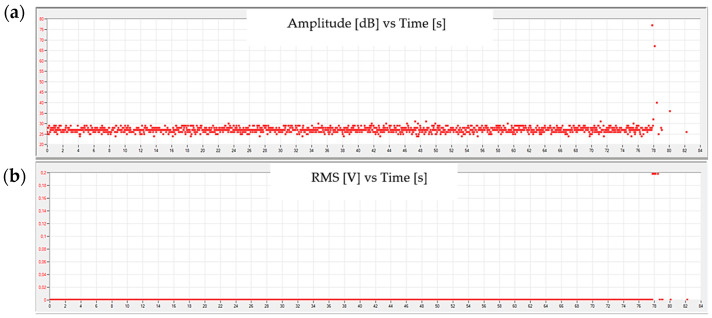
Sample graphs showing the change in (**a**) amplitude and (**b**) RMS of the signal as a function of time for a pure resin sample.

**Figure 7:**
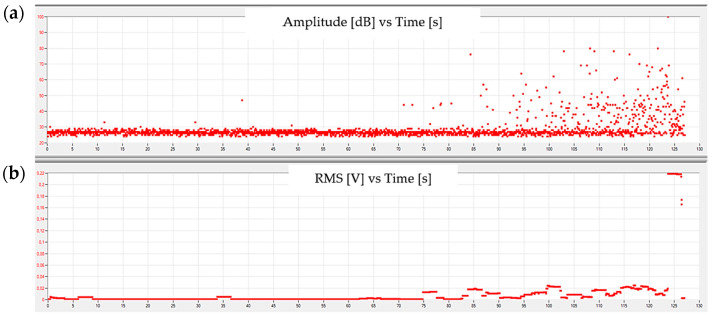
Sample graphs showing the change in (**a**) amplitude and (**b**) RMS of the signal as a function of time for a composite sample.

Figure 6 and Figure 7 show example graphs of the amplitude and root mean square (RMS) of the measured AE signal changes of the signal recorded by the measuring system. 

The conducted research clearly shows the nature of the researched materials. In the case of pure resin samples, typical failures of brittle materials can be observed. For studied case, the increase in the RMS value took place immediately before the sample fracture-about 0.1 s before recording the maximum amplitude. The amount of released energy can be proved by the increase in RMS of the signal (over 300 times) in comparison to the previously recorded values. 

The composite sample was of a different nature. Changes in signals generated by elastic–plastic deformation and matrix fracture, as well as fibre breakage, are visible. The analysis of acoustic emission signals recorded during the tests helps to determine the values of amplitudes characteristic for the destruction mechanisms of the considered composite materials. Signals with an amplitude in the range of 30–41 dB may indicate elastic–plastic deformation of the matrix. Signals with an amplitude in the range of 42–50 dB indicate matrix cracks with the accompanying phenomenon of fibre delamination. Signals with amplitudes greater than 50 dB indicate fibre breakage. 

Related to completely different structure of composite comparing to pure resin, the RMS change clearly indicates the complexity of the failure process related to the matrix and reinforcement. In this case, the RMS value increased by about eight times at the time preceding the matrix fracture. An increase in RMS started about 5 s before the matrix fracture occurred, while decreased to previous level. This indicates an increase in stresses and an elasto-plastic deformation. The fracture is a short-term phenomenon and is characterized by a sudden increase in amplitude (rapid energy release) with a simultaneous decrease in RMS to the acoustic noise level recorded before the stress increase. Similarly, fibre breakage is accompanied by RMS increasing (over 30 times) 3 s before the maximum amplitude.

**Figure 8:**
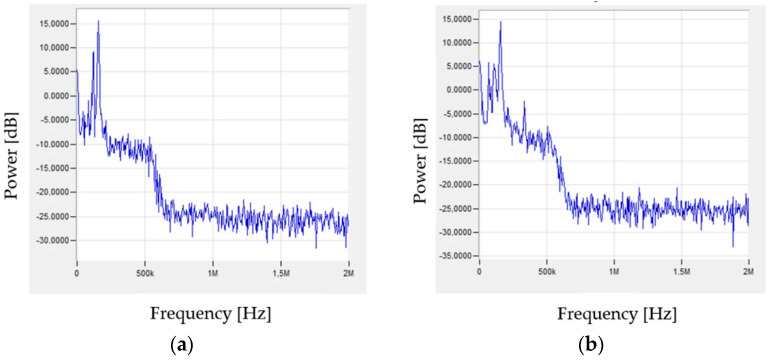
Examples of signals, after FFT analysis, characteristic for matrix deformation: (**a**) sample of pure resin, (**b**) composite.

**Figure 9:**
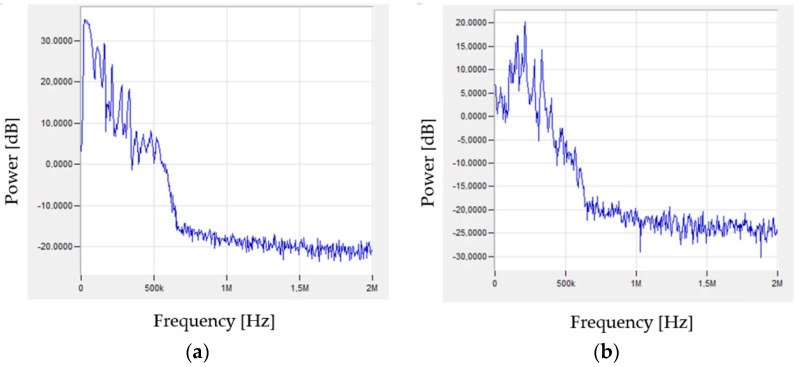
Examples of signals, after FFT analysis, characteristic for cracking of matrix: (**a**) sample of pure resin, (**b**) composite.

**Figure 10:**
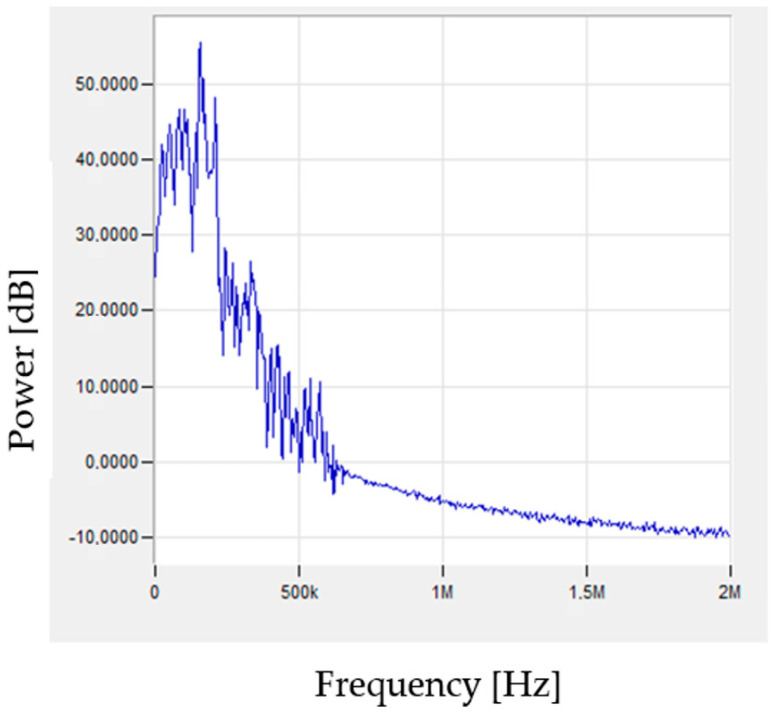
Example of signal, after FFT analysis, characteristic for cracking of fibres.

The signals generated during the tests were subjected to fast Fourier transform (FFT) analysis. Characteristic signals corresponding to specific stresses that may indicate individual failure mechanisms of the tested composites are shown in Figure 8, Figure 9 and Figure 10. 

A similar signal character can be observed in the case of samples from the pure resin and reinforced samples. Both the matrix deformation and cracking have a similar frequency spectrum in both considered cases. The delamination by decohesion of the matrix from the fibres can also be interpreted as the initiation of matrix fractures. The similar nature of the matrix cracks and delamination manifests itself in the generation of signals of a similar nature and frequencies. While the nature of the signal is similar, the amplitude values of the recorded signals are obviously different due to the amount of energy released during the destruction of individual components of the composite material. In the case of a pure resin sample, the sample breaks completely, as opposed to the reinforced sample, where the thin matrix layers between the fibres break. Sudden phenomena, which are undoubtedly cracks in both the matrix and the fibres, generate additional signals of higher frequencies (Figure 9 and Figure 10) compared to the elasto-plastic deformation (Figure 8). Table 2 presents the results obtained during research.

**Figure 11:**
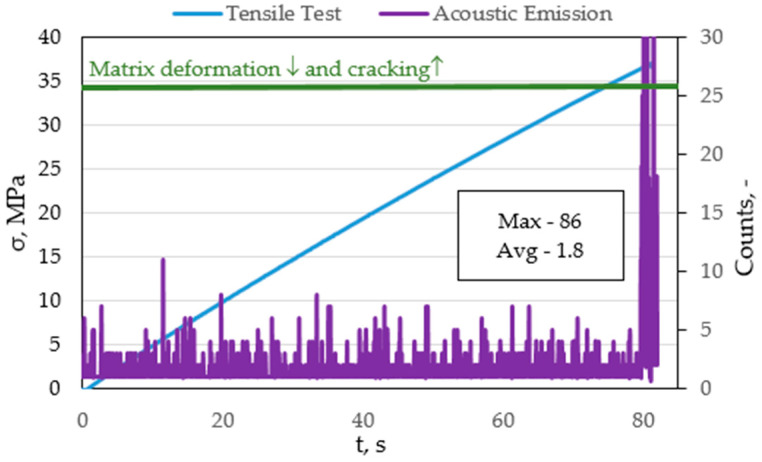
Graph of the counts plotted on the graph of tensile versus time for a selected sample based on pure polyester resin.

The article [14] presents the results for CFRP composites. The warp deformation also occurs in the range of 30–40 dB. Damage propagation occurs, as well as matrix cracking at values from 40–80 dB. It was determined that delamination occurs at amplitudes above 70 dB. The results obtained in these tests for composites reinforced with glass fibre indicate a similar nature of destruction despite the use of carbon fibre reinforcement and the use of other types of loads. Figure 11 shows the graph of the number of events plotted on the stretch diagram as a function of time.

**Figure 12:**
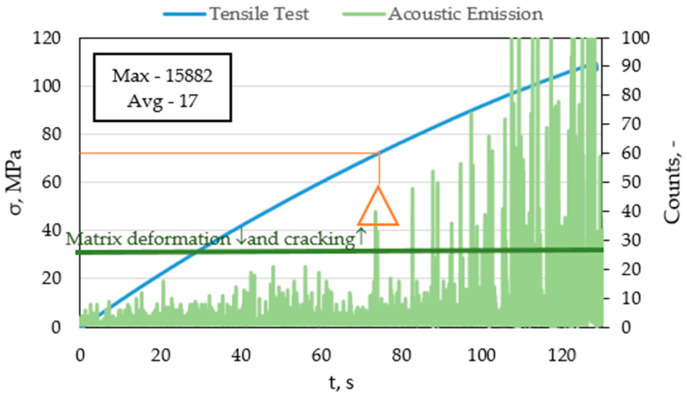
Graph of the counts plotted on tensile versus time plots for a selected FRP sample.

In the graph, the green line marks the range to which we deal with deformation and cracking of the matrix (~35 MPa). Figure 12 shows a graph of counts plotted on a tensile graph as a function of time for a selected FRP sample. 

Analyzing the graph in Figure 12 for a selected FRP sample, it can be concluded that until the stress of about 70 MPa, there are no significant changes in tested material. The events above the matrix fracture line, represent slight structural changes in the composite material. After exceeding 70 MPa, the number of counts increases steadily, matrix fractures, delamination, fibre cracking, etc. occur in the composite material.

Exemplary samples were selected for the comparative analysis, while the average values obtained are presented in Table 3.

## 4. Discussion

Supplementing the measuring system with the acoustic emission system helped to unequivocally determine the boundary stress levels for individual stages of destruction process. During the tests, various AE parameters were recorded: RMS, amplitude, counts, duration time, rise time, and energy. The parameter of the greatest diagnostic importance was the signal amplitude and the number of events. During the tests of the base sample (pure resin), the nature of the cracking was determined and then compared with the composite sample. For this purpose, the frequency spectra of the signals subjected to the FFT analysis were compared. On this basis, it was possible to determine the signals generated by the fracture of the matrix of the composite.

The research results showed that signals with an amplitude in the range of 30–41 dB may indicate plastic deformation of the matrix. This corresponds to the stress of the tested material up to the level of about 70 MPa. Signals with an amplitude in the range of 42–50 dB are characteristic of matrix cracks with the accompanying phenomenon of fibre delamination and occur at stresses in the range of 70–80 MPa. Signals with amplitudes greater than 50 dB indicate fibre breakage. In this case, the load on the test samples exceeded 80 MPa. When this stress is exceeded, the process of destroying both the fibres and the matrix of the composite takes place. In the article [14], the researchers obtained similar ranges of changes in AE signals in the bending tests of CFRP composites, which may indicate individual stages of the destruction process. Based on the obtained results and comparative analysis of acoustic emission parameters for samples based on polyester resin (Base), and also FRP, it can be concluded that they differ significantly. Significant changes in the composite material, which has a tensile strength of about 120 MPa, take place at a stress of about 80 MPa. It was determined that the deformation and fracture of the matrix in the case of samples made of pure polyester resin was in the range of 30–33 dB, while samples with FRP generated a signal with an amplitude in the range of 41–50 dB. This is due to the fact that, in FRP samples, the matrix thickness is many times smaller and is related to the complex structure and the adhesion between the matrix and the reinforcement. In the case of the number of counts, there is also a significant difference between the baseline samples and the FRP. The mean number of events for the baseline samples is around 3 and, for the FRP samples, it is around 16. For RMS, only a difference is observed at the mean maximum values and there is a 17-fold increase in the parameter. This is due to the fact that the RMS acoustic emission signal energy value representing the signal energy is not a tool for the analysis of fast-varying signals. However, when analysing this parameter, one can clearly see the difference in the nature of the destruction processes of the tested materials. No change in the RMS value of the pure resin occurs until the resin breaks. In the case of composite samples, changes in the RMS value are visible, which indicate the cracking of the matrix and reinforcement fibres. The event duration parameter was also helpful in the interpretation of the results. Signals of relatively short duration (in the range of 100–300 µs) suggest sudden phenomena, such as matrix or fibre cracking. Long times are characteristic of background noise or continuous phenomena and, therefore, could be eliminated relatively easily from consideration. All observed differences depend on the type of material. Reinforcement in the form of fibres changes the character of cracking, from brittle to elastic–plastic, and thus increases the value of most of the recorded parameters. In the article [14], the researchers obtained similar ranges of changes in AE signals in the bending tests of CFRP composites, which may indicate individual stages of the destruction process.

## 5. Conclusions

1. The destruction mechanism of composite materials consists of several stages, e.g., matrix fracture, fibre fracture, fracture at the fibre–matrix interface, and delamination. The registration of the change in force and elongation, i.e., the parameters obtained from the testing machine, are not sufficient to determine the previously mentioned individual stages of the destruction process.
2. Based on the test results, the permissible stress was determined; when exceeded, the mechanisms of damage to the structure of composite materials accumulate. This stress limit for the tested material is 70 MPa.
3. Knowing the safety stress limit can be very helpful in design work with FRP composite material. As the use of composite materials continues to increase in industries around the world, the research proposed in the article may be of great help.
4. Materials subjected to variable loads should be tested and their fatigue resistance determined, which is planned as the next stage of research.

## Figures and Tables

**Table 1:** Properties of resin and reinforcement used in the manufacturing process.

Parameter	UTS, MPa	E, MPa	EL, %
Polyester resin	70	4300	2
Fibre glass	2900–3600	72,000	3.37
**UTS**—Ultimate tensile strength, MPa; **E**—Young Modulus, MPa; **EL**—elongation, %			

**Table 2:** Summary of the analysis of the materials degradation.

Signal Type	Amplitude, dB	Stress, MPa
Matrix deformation	31–41	10–40
Cracking of matrix, delamination of fibres	42–50	38–50
Cracking of fibres	>50	51–110

**Table 3:** Summary of the obtained results.

AE Parameter		Base	FRP
Average Amplitude	dB	26	28
Maximum Amplitude	dB	33	100
Average Counts	-	3.16	16.52
Maximum Counts	-	86	1252
Average RMS	V	0.0006	0.0006
Maximum RMS	V	0.0008	0.1736
Average Duration	μs	381	464
Maximum Duration	μs	6946	130,920

## Data Availability

Data available on request due to restrictions eg privacy or ethical. The data presented in this study are available on request from the corresponding author. The data are not publicly available due to its huge amount.

## References

[1] Krolikowski W. (2012). Srtuctural polimer composites [in Polish]. Polimerowe Kompozyty Konstrukcyjne.

[2] Mrówka M., Woźniak A., Nowak J., Wróbel G., Sławski S. (2021). Determination of Mechanical and Tribological Properties of Silicone-Based Composites Filled with Manganese Waste. Materials.

[3] Mrówka M., Woźniak A., Prężyna S., Sławski S. (2021). The Influence of Zinc Waste Filler on the Tribological and Mechanical Properties of Silicone-Based Composites. Polymers.

[4] Panasiuk K., Dudzik K., Hajdukiewicz G. (2021). Acoustic Emission as a Method for Analyzing Changes and Detecting Damage in Composite Materials During Loading. Arch. Acoust..

[5] Kyzioł L., Panasiuk K., Hajdukiewicz G., Dudzik K. (2021). Acoustic Emission and K-S Metric Entropy as Methods for Determining Mechanical Properties of Composite Materials. Sensors.

[6] Nikbakht M., Yousefi J., Hosseini-Toudeshky H., Minak G. (2017). Delamination evaluation of composite laminates with different interface fibre orientations using acoustic emission features and micro visualization. Compos. Part B Eng..

[7] Saeedifar M., Fotouhi M., Ahmadi Najafabadi M., Hosseini Toudeshky H., Minak G. (2016). Prediction of quasi-static delamination onset and growth in laminated composites by acoustic emission. Compos. Part B Eng..

[8] Wojas G. (2015). Quality issues of non-destructive testing—General requirements for the competence of testing laboratories [in Polish: Zagadnienia jakości badań nieniszczących. Wymagania ogólne w zakresie kompetencji laboratoriów badawczych]. Przegląd Spaw..

[9] Ono K. (2019). Structural Health Monitoring of Large Structures Using Acoustic Emission–Case Histories. Appl. Sci..

[10] Dudzik K., Labuda W. (2020). The Possibility of Applying Acoustic Emission and Dynamometric Methods for Monitoring the Turning Process. Materials.

[11] Rui G., Guijun X., Chenggao L., Xiangyu H., Meiyin X. (2021). Effect of fibre hybridization types on the mechanical properties of carbon/glass fibre reinforced polymer composite rod. Mech. Adv. Mater. Struct..

[12] Guijun X., Rui G., Chenggao L., Bin H. (2021). Effects of rod size and fibre hybrid mode on the interface shear strength of carbon/glass fibre composite rods exposed to freezing-thawing and outdoor environments. J. Mater. Res. Technol..

[13] McCrory J.P., Al-Jumaili S.K., Crivelli D., Pearson M.R., Eaton M.J., Featherston C.A., Guagliano M., Holford K.M., Pullin R. (2005). Damage classification in carbon fibre composites using acoustic emission: A comparison of three techniques. Compos. Part B Eng..

[14] Šofer M., Cienciala J., Fusek M., Pavlíček P., Moravec R. (2021). Damage Analysis of Composite CFRP Tubes Using Acoustic Emission Monitoring and Pattern Recognition Approach. Materials.

[15] Zhuang X., Yan X. (2006). Investigation of damage mechanisms in self-reinforced polyethylene composites by acoustic emission. Compos. Sci. Technol..

[16] Yu Y.-H., Cho J.-H., Kweon J.-H., Kim D.-H. (2006). A study on the failure detection of composite materials using an acoustic emission. Compos. Struct..

[17] Kocich R., Cagala M., Crha J., Kozelsky P. Character of acoustic emission signal generated during plastic deformation. Proceedings of the 30th European Conference on Acoustic Emission Testing & 7th International Conference on Acoustic Emission.

[18] Yu F.-M., Okabe Y., Wu Q., Shigeta N. (2016). A novel method of identifying damage types in carbon fibre-reinforced plastic cross-ply laminates based on acoustic emission detection using a fibre-optic sensor. Compos. Sci. Technol..

[19] Guo Y., Zhu S., Chen Y., Li D. (2019). Analysis and Identification of the Mechanism of Damage and Fracture of High-Filled Wood Fibre/Recycled High-Density Polyethylene Composites. Polymers.

[20] De Rosa I.M., Santulli C., Sarasini F. (2009). Acoustic emission for monitoring the mechanical behaviour of natural fibre composites: A literature review. Compos. Part A Appl. Sci. Manuf..

[21] Saeedifar M., Najafabadi M.A., Zarouchas D., Toudeshky H.H., Jalalvand M. (2018). Clustering of interlaminar and intralaminar damages in laminated composites under indentation loading using Acoustic Emission. Compos. Part B Eng..

[22] Sobhani A., Saeedifar M., Najafabadi M.A., Fotouhi M., Zarouchas D. (2018). The study of buckling and post-buckling behavior of laminated composites consisting multiple delaminations using acoustic emission. Thin-Walled Struct..

[23] Guo Y., Zhu S., Chen Y., Liu D., Li D. (2019). Acoustic Emission-Based Study to Characterize the Crack Initiation Point of Wood Fibre/HDPE Composites. Polymers.

[24] Oskouei A.R., Zucchelli A., Ahmadi M., Minak G. (2011). An integrated approach based on acoustic emission and mechanical information to evaluate the delamination fracture toughness at mode I in composite laminate. Mater. Des..

[25] Fotouhi M., Pashmforoush F., Ahmadi M., Refahi Oskouei A. (2011). Monitoring the initiation and growth of delamination in composite materials using acoustic emission under quasi-static three-point bending test. J. Reinf. Plast. Compos..

[26] Fotouhi M., Sadeghi S., Jalalvand M., Ahmadi M. (2017). Analysis of the damage mechanisms in mixed-mode delamination of laminated composites using acoustic emission data clustering. J. Thermoplast. Compos. Mater..

